# Emergency preparedness and readiness; anticipating the need for rehabilitation

**DOI:** 10.2471/BLT.22.289085

**Published:** 2022-10-03

**Authors:** Pete Skelton, Flavio Salio, Nedret Emiroglu

**Affiliations:** a Rehabilitation Programme, World Health Organization, Avenue Appia 20, 1211 Geneva 27, Switzerland.; b Country Readiness Strengthening, Health Emergencies, World Health Organization, Geneva.

Since the beginning of this decade, all countries have faced serious health emergencies, whether due to wars, earthquakes, cyclones, floods, industrial explosions, fires or the global coronavirus disease 2019 (COVID-19) pandemic. These events tragically continue to kill many people; however, the number of people experiencing injury or impairment that require acute and ongoing rehabilitation is far higher – and increasing. Between 2010 and 2019, available data show the number of people injured in disasters (6.7 million) was four times more than those killed (1.5 million).^1^

We do not have a true picture of rehabilitation needs in emergencies due to a lack of research and little or no monitoring of health outcomes in emergencies other than mortality. However, health emergencies create significant surges in pressing and often unmet rehabilitation needs. Improved emergency, surgical and critical care are also now saving more lives, and with more survivors, rehabilitation needs may further increase. Early rehabilitation in emergencies is therefore essential: it can maximize the impact of medical and surgical interventions; speed up recovery; optimize functioning; and enhance quality of life of survivors.^2^ Additional health service benefits include reduced length of stay in hospital, decreased readmissions, and the prevention of costly and potentially fatal complications.^3^

When rehabilitation needs are not quickly addressed, individuals, their families and communities face far-reaching consequences. For example, a lack of early rehabilitation for a patient with burns after a tanker explosion may result in preventable contractures and a subsequent loss of function and income. A child who loses a limb during a conflict and does not have access to early rehabilitation may develop complications or not receive a prosthesis, and therefore find their participation in education restricted. A patient hospitalized with COVID-19 leaves hospital still dependent on their family for care due to a low prioritization of rehabilitation. In each of these examples, people directly affected by emergencies suffer unnecessarily and have a suboptimal recovery. Moreover, their families and communities are affected as well.

The origins of modern rehabilitation are in emergencies; it developed as a treatment strategy in part due to a surge in needs as a result of war and polio epidemics in the early 20th century. More recently, key humanitarian operational^2^ and clinical guidelines^4^^,^^5^ have included rehabilitation as an essential component of care. Such guidelines include the World Health Organization (WHO) *Emergency Medical Teams: minimum technical standards and recommendations for rehabilitation*.^6^

Despite its origins, and although many response guidelines exist, rehabilitation is rarely prioritized early in emergencies and a misconception exists that rehabilitation comes later in the continuum of care. Too often, responders cite the humanitarian imperative, with the priority being to save lives. However, this conception is a false dichotomy: the humanitarian imperative includes the prevention or alleviation of human suffering, in which rehabilitation plays a critical role.

Response challenges are not just attitudinal; existing rehabilitation services are often under-resourced, poorly integrated into health systems and quickly overwhelmed in emergencies. Such underlying weaknesses and barriers underscore the importance of preparedness to best utilize scarce rehabilitation resources.

Research on health system preparedness is lacking, specifically on the status and impact of rehabilitation preparedness. Notwithstanding, rehabilitation appears to be almost universally absent from health emergency preparedness. Data from WHO’s work to strengthen rehabilitation in health systems showed that only one of 19 low- and middle-income countries involved had integrated rehabilitation into health emergency preparedness. The example of Nepal is a key outlier, where specific rehabilitation preparedness, including training of staff, development of protocols and stockpiling of equipment, improved the ability of service providers to respond.^7^ Evidence from responses in high-income settings indicate that the situation is not much different there – a lack of rehabilitation preparedness has repeatedly been a key barrier to an early effective response – with the example of Japan being one positive exception.^8^

An overall approach to rehabilitation in health systems strengthening will improve access to rehabilitation in emergencies, but specific steps must be taken to better prepare rehabilitation services. Preparedness must also be all-hazard, that is, with plans to maintain essential services as well as specific considerations for hazards that may cause a surge in rehabilitation needs, including but not limited to natural hazards such as earthquakes, cyclones, floods and disease outbreaks; societal hazards, such as conflict and terrorism; and technological hazards such as major explosions, fires and chemical or radiological events. The COVID-19 pandemic has provided a stark example of the need for rehabilitation in outbreak preparedness. Rehabilitation services were needed for acute hospitalized patients, patients with post-intensive care syndrome and those with post COVID-19 condition.^9^ However, a WHO survey of 105 countries between March and June 2020 showed that rehabilitation was among the most disrupted services in the early months of the pandemic.^10^ Polio, measles, severe acute respiratory syndrome, Middle East respiratory syndrome, diphtheria and Ebola virus disease outbreaks^11^ have also generated acute or post-acute rehabilitation needs. Eight of the nine current WHO priority diseases of epidemic potential are likely to generate such needs.

WHO’s Health Emergency and Disaster Risk Management Framework,^12^ developed in 2019 to create a paradigm shift and comprehensive approach to managing health emergencies, offers a path forward to strengthen rehabilitation preparedness. Using the framework’s 10 components as well as lessons learnt from recent responses and existing Inter-Agency Standing Committee Guidelines,^13^ we argue that specific steps must be taken to integrate rehabilitation. Further health policy and systems research would also inform decision-making regarding how best to integrate rehabilitation into emergency preparedness.

## Health systems

Those working on health systems preparedness must ensure that rehabilitation considerations are included in national or subnational risk assessments, planning and coordination.^13^ Rehabilitation leadership must be included in preparedness planning and in response coordination,^7^ with a dedicated rehabilitation role in health emergency operations centres. Key rehabilitation services must be mapped^2^ and emergency referral pathways developed.^2^^,^^7^^,^^13^ Policy and legislation must enshrine the inclusion of rehabilitation in emergency management, while information management systems must be able to track patients requiring rehabilitation follow-up and monitor functioning outcomes.^14^ Monitoring and evaluation frameworks and reviews must look beyond immediate mortality to consider the medium and longer-term outcomes of patients.^14^ Governments and donors must make financial resources available for rehabilitation preparedness^13^ and response.

## Health services

Service leads must examine critical gaps; rehabilitation surge support mechanisms should be built,^7^ including the use of rosters and the integration of rehabilitation professionals into Emergency Medical Teams.^6^ Difficult decisions must be taken about which rehabilitation services to prioritize in an emergency, and which can be diverted.^9^ Plans to adapt essential services to ensure continuation and service coverage of hard-to-reach populations should be developed.^9^^,^^13^ Adapted clinical protocols should be created,^13^ along with condition-specific patient education resources for conditions that are likely to surge.^7^ The rehabilitation workforce must have identified roles in response and be trained to perform these.^13^^,^^15^ Health service simulation exercises should include rehabilitation considerations and rehabilitation teams. Stockpiles of essential rehabilitation equipment and assistive products should be developed,^7^^,^^13^ and surge supply chains tested. Rehabilitation services, particularly those that support patients with complex health conditions, should consider integrating risk communication and personal preparedness measures into their work with patients to reduce the risks they face in emergencies, and by promoting community level preparedness.^16^

## Health facilities

Rehabilitation infrastructure must be safe and accessible,^12^^,^^13^ with regularly rehearsed evacuation procedures^17^ that are specific to the hazards the facility may face. Essential rehabilitation equipment such as crutches and wheelchairs must be locally stockpiled.^13^ The role of any rehabilitation space, including inpatient beds, must be integrated into response planning, and plans developed to create additional capacity in the event of a surge in need.^5^^,^^13^ Facilities must have their own plans to train, mobilize and support their rehabilitation workforce.

## Individual professionals

Rehabilitation professionals must understand the potential hazards they face and have personal and professional preparedness plans in place.^15^ They may also play a critical role in advocating for greater preparedness in their workplace, and by engaging with rehabilitation, health and health emergency leaders to promote the inclusion of rehabilitation in all-hazard emergency preparedness.

## Conclusion

The steps proposed above would help ensure that patients receive the early and ongoing rehabilitation needed in emergencies, and that the long-term outcomes of those affected are not neglected. Importantly, many of these steps support both emergency preparedness and the continued strengthening of rehabilitation within the health system. WHO will contribute to this work by developing a policy brief and practical toolkit to support the integration of rehabilitation into emergency preparedness and response. The new WHO-hosted World Rehabilitation Alliance, which focuses on promoting rehabilitation as an essential health service, will also include emergency preparedness as a key advocacy workstream to further this agenda.
